# 

**Published:** 1860-03

**Authors:** 


### Medical Association of Georgia.

The annual meeting of the Medical Association of Georgia, will take place in the city of Rome, on the second Wednesday in April, 1860. We hope to see a large attendance of the profession. Our brethren of Rome, we know will be pleased to see the Medical men from every quarter of the State. It is time we think, that our State association, and State Institutions generally, should take precedence of those that are National, and as a matter of course, are controlled by northern influence.

